# A study to reduce readmissions after surgery in the Veterans Health Administration: design and methodology

**DOI:** 10.1186/s12913-017-2134-2

**Published:** 2017-03-14

**Authors:** Laurel A. Copeland, Laura A. Graham, Joshua S. Richman, Amy K. Rosen, Hillary J. Mull, Edith A. Burns, Jeff Whittle, Kamal M. F. Itani, Mary T. Hawn

**Affiliations:** 1 Veterans Affairs: VA Central Western Massachusetts Healthcare System, Leeds, MA USA; 2 Texas A & M Health Science Center, College of Medicine, Temple, TX USA; 3 Department of Psychiatry, UT Health Science Center San Antonio, San Antonio, TX USA; 4 Veterans Affairs, Birmingham VAMC, Birmingham, AL USA; 5 Veterans Affairs, Center for Healthcare Organization and Implementation Research (CHOIR), VA Boston Healthcare System, Boston, MA USA; 6 Department of Surgery, Boston University School of Medicine, Boston, MA USA; 7 VA Boston Healthcare System, Boston, MA USA; 8 Harvard School of Medicine, Cambridge, MA USA; 9 Veterans Affairs, Milwaukee VAMC, Milwaukee, WI USA; 10 Veterans Affairs, Palo Alto VAMC, Palo Alto, CA USA; 11 Department of Surgery, Stanford University School of Medicine, Palo Alto, CA USA

**Keywords:** Comorbidity, Methods, Quality of care, Surgical procedures, Operative, Veterans

## Abstract

**Background:**

Hospital readmissions are associated with higher resource utilization and worse patient outcomes. Causes of unplanned readmission to the hospital are multiple with some being better targets for intervention than others. To understand risk factors for surgical readmission and their incremental contribution to current Veterans Health Administration (VA) surgical quality assessment, the study, Improving Surgical Quality: Readmission (ISQ-R), is being conducted to develop a readmission risk prediction tool, explore predisposing and enabling factors, and identify and rank reasons for readmission in terms of salience and mutability.

**Methods:**

Harnessing the rich VA enterprise data, predictive readmission models are being developed in data from patients who underwent surgical procedures within the VA 2007–2012. Prospective assessment of psychosocial determinants of readmission including patient self-efficacy, cognitive, affective and caregiver status are being obtained from a cohort having colorectal, thoracic or vascular procedures at four VA hospitals in 2015–2017. Using these two data sources, ISQ-R will develop readmission categories and validate the readmission risk prediction model. A modified Delphi process will convene surgeons, non-surgeon clinicians and quality improvement nurses to rank proposed readmission categories vis-à-vis potential preventability.

**Discussion:**

ISQ-R will identify promising avenues for interventions to facilitate improvements in surgical quality, informing specifications for surgical workflow managers seeking to improve care and reduce cost. ISQ-R will work with Veterans Affairs Surgical Quality Improvement Program (VASQIP) to recommend potential new elements VASQIP might collect to monitor surgical complications and readmissions which might be preventable and ultimately improve surgical care.

## Background

Readmissions following hospital discharge result in increased costs, resource utilization and worse patient outcomes [1, 2]. Reducing avoidable readmission is therefore a quality improvement goal. As part of the Affordable Care Act (ACA), excessive rates of readmissions now result in reduced hospital Medicare reimbursement, affecting up to two-thirds of US hospitals in the private and federal sectors [3]. The Veterans Health Administration (VA) is not yet directly penalized for readmissions by ACA or VA-specific payment rules. However, it is under increased pressure to become more transparent in its processes of care. VA makes public both facility and nationwide readmission rates each quarter. Similar to the private sector, the VA now reports outcome measures, including readmissions, on the Centers for Medicare and Medicaid Services Hospital Compare website.

Readmission following admission for specific medical conditions has been the focus of many studies, but much less is known about factors associated with surgical readmission. In contrast to medical admissions, index surgical admissions are usually planned, patients are screened for risk factors prior to admission, and surgical procedures have well-known and expected complication rates. Thus, correlates of unplanned readmission after surgery may differ markedly from those of medical readmissions. Primary among these are older age and comorbidity burden, [4–6] poor social or caregiver support, [7] more numerous prescription medications at discharge, [6] previous hospitalizations, [8] and functional disability [9]. Predisposition to readmission also varies by surgical procedure type: colorectal surgeries have relatively high rates of surgical site infection and gastrointestinal complications, [10] abdominal procedures are associated with dehydration and ileus, [4] and vascular surgeries are associated with readmission for bleeding, groin wound complication and wound debridement [11].

To date, much of our knowledge regarding the quality of surgical care in the VA comes from the Veterans Affairs Surgical Quality Improvement Program (VASQIP) which has been adopted by portions of the private sector [12]. VASQIP assesses complications that occur after surgery but is not an exhaustive source of post-operative complications; readmission may be a proxy for other post-operative factors not currently assessed.

To address the knowledge gap we have initiated a study, “Improving Surgical Quality: Readmission (ISQ-R)”, a mixed methods study comprising retrospective analysis and prospective surveys regarding surgeries with consistently high rates of unplanned readmissions: colorectal, thoracic and vascular procedures [13]. ISQ-R will (a) develop risk prediction models of 30-day readmission and classify reasons for readmission in retrospective data; (b) prospectively collect patient psychosocial factors at discharge and correlate with 30-day readmission; and (c) assemble a Delphi panel of experts to rank the identified reasons for readmission with respect to their potential preventability and suitability for use as care quality measures.

## Methods

### Study design and aims

**Fig. 1:**
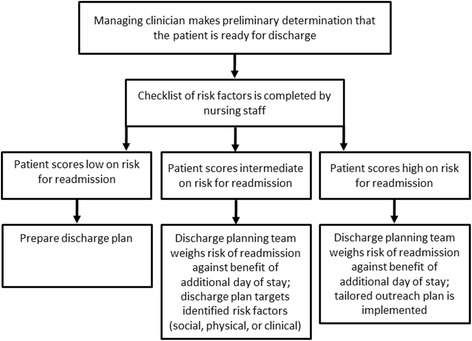
Schematic of implementation of the new readmission risk tool in clinical practice

An overview of the study aims and methods is presented graphically in Table 1. The first ISQ-R aim is to evaluate the contribution of patient, procedure, postoperative and system factors to unplanned readmission within 30 days of hospital discharge following major surgery (open procedures for definitive treatment, with at least 2-day stay), [14] and use these data to develop and validate a readmission risk prediction model that can be used real-time (see Fig. 1). We will develop a classification of readmission reasons and explore processes of care linked with readmission using a cohort of VASQIP-assessed major surgeries from 2007 to 2012. Reasons for readmission will be identified by International Classification of Disease, 9^th^ Edition (ICD9) diagnosis codes and categorized by etiology [15].

**Table 1:** Overview of ISQ-R study aims and methods

Aim	Methods
1 Evaluate contribution of patient, procedure, postoperative and systems factors following major surgery (with minimum 2-day stay) to develop and validate a readmission risk prediction model. Classify reasons for readmissions and related processes of care.	Acquire and merge retrospective data from the VA Surgical Quality Improvement Program for all assessed surgeries between October 1, 2007 and September 30, 2014
	Investigate predictors of 30-day unplanned readmission following surgery using logistic regression
	Develop a risk prediction tool for 30-day unplanned readmission following surgery
	Determine readmission reason categories from primary ICD9 diagnosis codes
	Report findings to National Surgery Office Advisory Board
2 Assess potential patient factors not currently collected by VASQIP for association with readmission	Develop and pilot a prospective survey to assess patient psychosocial factors at discharge
	Recruit 800 surgical patients from four (4) VA sites distributed across the nation
	Administer prospective survey prior to discharge and follow patients for 30 days post-discharge to assess readmission
	Assess the association between psychosocial factors not currently assessed in administrative data and 30-day unplanned readmission in the prospective cohort
	Further explore predictors of 30-day unplanned readmission following surgery using psychosocial factors
3 Rank reasons for readmission based on Aim 1 and Aim 2 and assess for potential preventability and appropriateness for classification as a measure of surgical quality.	Develop Delphi process form using readmission reasons defined in Aim 1
	Convene Delphi panel participants
	Rank readmission reasons as (1) potentially preventable and (2) appropriate measures of surgical quality
	Report findings to National Surgery Office Advisory Board

Our second aim is to assess potential patient factors not currently collected by VASQIP and analyze their association with readmission using surveys at four VA facilities. The readmissions literature on surgical populations hypothesizes that social support and caregiver factors may contribute to such events [16, 17]. To frame our study we used the Andersen Behavioral Model for Healthcare Utilization, a model frequently used to explain variation in health services utilization [18]. Andersen proposes three main classes of factors: (1) Need, (2) Predisposing and (3) Enabling. Andersen’s Behavioral Model was designed to describe factors explaining healthcare use and equitable access. It has been applied to both acute care and emergency department utilization [19, 20]. Need is the primary and most proximate predictor of healthcare utilization, operationalized by surgery and comorbidity. Predisposing factors (i.e., age, education, self-efficacy, self-management, cognitive function, mood) and enabling factors (i.e., transportation, caregiver status, access to primary care) affect the likelihood of utilizing healthcare and further explain or predict variation in utilization. In the case of 30-day readmissions, predisposing factors expected to correlate most strongly with post-operative readmissions include poor self-efficacy and poor self-management skills while predominant enabling factors include insufficient caregiver help.

Our third aim is to rank reasons for readmission developed from Aims 1 and 2 for (a) potential preventability and (b) appropriateness as a measure of surgical quality. Using a Delphi process, our panel will rate reasons by potential preventability and potential to serve as a measure of surgical quality beyond the current VASQIP measures. The study was approved by the VA Central Institutional Review Board prior to initiation.

### Cohort

We will develop two study cohorts, one retrospective and one prospective. The retrospective cohort for Aim 1 will consist of patients with VASQIP-assessed major procedures in 2007–2012 with post-operative length of stay (LOS) of 2–30 days and alive at discharge [21]. This cohort will be linked with VA administrative data to examine predictors of readmission within VA [22, 23]. The prospective cohort will consist of patients in four VA facilities undergoing colorectal, thoracic or vascular surgeries over a 12-month period. We chose these surgeries as they have high rates of readmission; however, the reasons for readmission differ across groups. Colorectal cases are associated with more infectious and electrolyte disturbance reasons, whereas vascular and thoracic cases are associated with more cardiovascular and bleeding reasons. We will use this prospective cohort to validate the surgical readmission risk prediction model and model reasons for readmissions.

We addressed two major considerations when constructing our cohorts. First, what constitutes major surgery and second, why choose the 30-day timeframe for assessing readmission. There are no standard definitions as to what constitutes a major surgical procedure. Major surgical procedures are more likely to have post-operative complications, consume more resources and have a higher rate of readmissions directly linked to the surgical intervention. For the readmission risk prediction model, we are interested in modeling readmission after surgery rather than unplanned admission after an outpatient procedure. To remove most outpatient cases, we require a minimum of two postoperative hospital days. Most procedures that can be performed on an outpatient basis will have a LOS of zero or one (overnight observation). The 2-day requirement also excludes most diagnostic procedures, such as intra-operative arteriograms and cystoscopies. Furthermore, our cohort is derived from VASQIP, which preferentially assesses major surgeries. The 30-day timeframe is commonly used; we will explore shorter and longer periods for readmission for our patients undergoing colorectal, thoracic or vascular surgery.

### Data source: VASQIP

VASQIP began in 1991 to analyze risk-adjusted 30-day morbidity and mortality within VA [14, 21]. Of approximately 375,000 surgical procedures in the VA yearly, 150,000 are major surgeries [24]. Complete assessment of perioperative data is documented on ~100,000 cases per year using a defined sampling algorithm to ensure adequate representation of the breadth and complexity of procedures. Data are abstracted from computerized sources. On the 30^th^ postoperative day, outcomes are collected from charts, morbidity and mortality conferences and communication with patients.

VASQIP currently assesses 21 complications within 30 days postoperatively and monitors 62 pre-surgical clinical and laboratory and 12 intraoperative risk factors. The risk-adjusted data obtained from this program have been reported back to VA medical centers and led to improvements in morbidity and mortality rates [25]. We will use selected variables from VASQIP (see Table 2).

**Table 2:** Data variables and data sources

Component	Variable	Data Source
Pre-Admission		
Demographics	Sex, Race/Ethnicity	CDW Patient Tables
	Age	CDW Vital Status Files
Comorbidities	Functional Status, DNR Status	VASQIP
	History of: Angina, Congestive Heart Failure, Cerebrovascular Accident, Peripheral Vascular Disease, Cardiac Surgery, Pre-operative Coma, Impaired Sensorium, Ascites, Esophageal Varices, Bleeding Disorders, Disseminated Cancer, Steroid Use, RBC Transfusion, Wound Infection, Weight Loss, Pneumonia, Ventilator Dependence, Dialysis, Acute Renal Failure	
Social/Behavioral	BMI, >2 Drinks/Day in the 2 Weeks Before Admission, Pack-Years Smoking	VASQIP
	Marital Status	CDW Patient Table
	Pre-Admission Inpatient and ER Utilization	CDW Inpatient and Outpatient Tables
Preoperative Labs & Vitals	Albumin, Bicarbonate, Bilirubin, BUN, Calcium, Chloride, Serum Creatinine, Creatinine eGFR, Glucose, Hematocrit, Hemoglobin, INR, Potassium, Sodium, WBC	MCA Laboratory
	Systolic & Diastolic Blood Pressure, Pain, Pulse, Pulse Oximetry, Respiration Rate, Temperature	CDW Vital Signs
Hospital Factors	Index Hospitalization Facility	VASQIP
Operative		
Complexity	Urgent/Emergent status, Inpatient/Outpatient status, Operative Time, Intraoperative RBC transfusion, Wound Classification, work RVU	VASQIP
Other	Year of Surgery, ASA classification, Anesthesia technique	VASQIP
Post-Operative		
Postoperative Labs & Vitals	Albumin, Bicarbonate, Bilirubin, BUN, Calcium, Chloride, Serum Creatinine, Creatinine eGFR, Glucose, Hematocrit, Hemoglobin, INR, Potassium, Sodium, WBC	MCA Laboratory
	Systolic & Diastolic Blood Pressure, Pain, Pulse, Pulse Oximetry, Respiration Rate, Temperature	CDW Vital Signs
Pre-Discharge Complications	Cardiac Arrest, Myocardial Infarction, Coma, Cerebral Vascular Accident, Wound Disruption, Failure to Wean, Peripheral Nerve Injury, Acute Renal Failure, Organ/Space SSI, RBC Transfusion, DVT/Thrombophlebitis, Pneumonia, Pulmonary Embolism, Reintubation, Progressive Renal Insufficiency, Sepsis, Superficial Infection, Urinary Tract Infection, Deep Wound Infection	VASQIP
ICU Utilization	ICU visits during the index hospitalization	CDW Inpatient Tables
Other Postoperative	Postoperative Length of Stay, Number of Surgeries during Index Hospitalization	VASQIP
	Discharge Destination	CDW Inpatient Tables
Post-Discharge		
Discharge Characteristics	Care Coordination, Caregiver Accessibility,	CTM-15
	Discharge Destination, Functional Status at Discharge, Transportation	Discharge, Readmission, and Follow-Up Interviews
Discharge Complexity	Medications, Wound Care Instructions, Mobility	Discharge Interview
Patient Characteristics at Discharge	General Health	VR-12
	Cognitive Function	SBT
	Pain at Discharge	Visual Analogue Scale
Post-Discharge Clinic Utilization	VA Clinic Stops in the 30-days post-discharge	CDW Outpatient Tables
Post-Discharge Complications	Cardiac Arrest, Myocardial Infarction, Coma, Cerebral Vascular Accident, Wound Disruption, Peripheral Nerve Injury, Acute Renal Failure, Organ/Space SSI, RBC Transfusion, DVT/Thrombophlebitis, Pneumonia, Pulmonary Embolism, Reintubation, Progressive Renal Insufficiency, Sepsis, Superficial Infection, Urinary Tract Infection, Deep Wound Infection	VASQIP
		Medical Chart Abstraction
Other	Changes in: Care Coordination, Caregiver Accessibility, Transportation, Medications	Follow-up Interview
	Pain at Follow-Up/Readmission	Visual Analogue Scale
Outcome		
Inpatient Readmission	Inpatient Admission within 30-days Following Index Hospitalization Discharge	CDW Inpatient Tables
		Readmission Interview
Unplanned ER Admission	ER utilization within 30-days Following Index Hospitalization Discharge	CDW Outpatient Tables
		Readmission Interview

*CDW* VA’s Corporate Data Warehouse, *VASQIP* VA Surgical Quality Improvement Program, *MCA* Managerial Cost Accounting (a series of VA files), *CTM* Care Transitions Measures, *SBT* Short Blessed Test; *BMI* Body Mass Index, *DNR* Do Not Resuscitate; *ER* Emergency Room, *BUN* Blood Urea Nitrogen, *eGFR* Estimated Glomerular Filtration Rate, *INR* International Normalized Ratio, *WBC* White Blood Cell, *RBC* Red Blood Cell, *RVU* Relative Value Unit, *ASA* American Society of Anesthesiologists Physical Status Classification

### VA administrative data

Administrative data in the VA’s centralized data repositories reliably capture all inpatient and outpatient healthcare encounters [26, 27]. Inpatient files include details of the stay: admission, discharge, diagnoses, procedures, transitions between bed sections (e.g., from surgical intensive care unit (ICU) to general medical), and provider types. Outpatient files document post-discharge follow-up care and emergency department visits. Files include age, gender, marital status, race/ethnicity, laboratory results, vital signs and pharmacy fills. Dates of death come from the VA Mini-Vitals, derived via validated algorithm with estimated 98% sensitivity from four sources: VA inpatient records, Veteran death benefits claims, Social Security and Medicare files [28].

VA patients may use non-VA healthcare. To examine the contribution of non-VA data to assessing readmissions, we used the Cardiac Stent Risk study [29] and identified 30-day readmissions within VA and Medicare/Medicaid files following non-cardiac surgery (see Table 3). Medicare/Medicaid Inpatient Institutional Claims identified readmissions to a non-VA facility following a VA surgery. While 30-day readmission rates were higher for veterans with Medicare/Medicaid versus without, the majority of patients (92%) were readmitted to a VA facility. Therefore, we will not use Medicare/Medicaid data in ISQ-R.

**Table 3:** Rate of readmission in VA and non-VA hospitals among veterans with and without medicare/medicaid coverage

	Total Surgeries		Total Readmissions		Readmissions within 30-days of Discharge					
					VA-Only		CMS-Only^a^		VA & CMS^a^	
	*N*	(%)	*N*	(%)	*N*	(%)	*N*	(%)	*N*	(%)
All Surgeries	22,648		3367	(14.9)	3231	(14.3)	88	(0.4)	48	(0.2)
No CMS Coverage	16,918	(74.7)	2285	(13.5)	2285	(13.5)	0	(0.0)	0	(0.0)
With CMS Coverage	5730	(25.3)	1082	(18.9)	946	(16.5)	88	(1.5)	48	(0.8)

*CMS* Centers for Medicare and Medicaid Services

^a^ CMS readmissions are defined as inpatient facility admissions in the 30 days following a VA surgery discharge

### Survey data

To address the predisposing and enabling factors targeted in Aim 2, the medical record alone would be insufficient. Therefore surveys will collect psychosocial factors from patients and caregivers. To improve the generalizability of our results, we will collect prospective data in four geographically distinct VA facilities: Birmingham, Boston, Milwaukee and Palo Alto.

The construct “discharge complexity” is one we felt was important although it is not routinely measured. Unable to identify any validated tool, we developed one specific to our study in preliminary work. The tool quantifies the complexity of the patient’s discharge instructions in the following domains: a) wound care; b) indwelling catheter/drain care; c) new ostomy (colorectal) or amputation (vascular); d) durable medical equipment; e) number of medications; and f) number of new medications. We pilot tested this tool in 30 patients, finalized the measure and will employ it in Aim 2 with 800 patients and their caregivers.

Other factors to be collected include activities of daily living, pain, perceived stress, depression, cognition and caregiver accessibility (see Table 4).

**Table 4:** Measures collected from patients undergoing colorectal, thoracic and vascular operations in four VA medical centers

RUG-ADL Assessment	The Resource Utilization Group – Activities of Daily Living Assessment measures functional status at discharge, readmission and at 30-day follow-up. This four-item validated questionnaire assesses a patient’s independence with mobility, toileting, transfer and eating [39].
Pain	The National Institutes of Health Numeric Rating Pain Scale assesses pain intensity at the time of discharge, readmission and 30-day follow-up [40].
Pain Meds	Total dose of pain medication administered 24 h (7 am-7 am) before discharge
Perceived Stress Scale	Cohen’s Perceived Stress Scale (PSS) is a 10-item scale that quantifies patient’s stress. The PSS has been shown to correlate with health behavior and health services utilization [41].
CES-D4	The Center for Epidemiologic Studies Depression Screen, 4-item version, assesses psychological distress at the time of discharge. The CES-D4 has been shown to be have a positive predictive value of 85% for depression in an older adult population [31].
MoCA	The Montreal Cognitive Assessment is a validated tool to assess a patient’s cognitive function. This tool has a positive predictive value of 89% for mild cognitive impairment (90% sensitivity; 87% specificity) when compared to clinical criteria supported by psychometric measures [42].
Caregiver Accessibility	These questions were guided by the literature around the immediacy and availability of the designated caregiver (i.e., does caregiver live with patient) [43, 44].
Transportation	Patient access to transportation and burden of transportation (number of post-operative visits and travel distance).
CTM-15, adapted	The Care Transition Measure is a 15-item scale that addresses the hospital’s efforts at care coordination at discharge. The survey also assesses patient self-efficacy in implementing the discharge plan. The tool was designed as a post-discharge, recall assessment [45]. We will adapt the tool: a) to assess these items on the day of discharge; b) to assess patient self-efficacy with wound, indwelling device, new ostomy or durable medical equipment, as applicable; and c) to assess patient understanding of whom and when to contact regarding warning signs or symptoms that may arise.
Institute for Healthcare Improvement Readmission Tool	This tool was developed by IHI as part of a conceptual roadmap to reduce avoidable re-hospitalizations by intervening at the system level. The tool will be adapted for surveying patients at readmission [46].
Brief Survey of Post-operative Care	Queries patient on unplanned emergency visit or readmission at an outside hospital; keeping post-operative appointments; difficulty getting medications filled (costs) and refilled (especially pain medication); receipt of home health or durable medical equipment.

### Study outcomes

The primary outcome of unplanned readmission will be reviewed for 30-day vs other possible time frames. In preliminary work, we estimated the median time to readmission as 10 days (IQR 4–18 days; unpublished data). Thus, 14 days is too short to capture many readmissions, while readmissions beyond 30 days are less likely to be linked to the index surgery. We will perform analyses to determine how shorter or longer periods may inform readmissions attributable to the surgical episode of care.

### Analysis plan

#### Model building for Aim 1

Logistic regression will be performed to investigate the predictors of 30-day readmission in VASQIP-assessed cases, evaluating the contribution of patient, procedure and system factors regardless of post-discharge mortality. To consider the relationship between death and readmission, sensitivity analyses will proceed in parallel to the main analyses using logistic regression to model the composite outcome of readmission or death, and Cox regression to model time to readmission considering death as a censoring event.

In building the predictive models we will consider surgical admissions to be nested within facilities and will use Generalized Estimating Equations (GEE) to account for clustering. Analyses will be repeated using generalized linear mixed models considering patients nested within facilities and surgeries within patients to check for consistency. We will estimate the intra-class correlation resulting from multiple observations per patient, and repeat models limiting cases to one surgery per patient to understand the impact of repeated surgeries.

The risk prediction models to identify at-risk patients in real time will operate at two distinct time points: prior to admission for surgery, and prior to discharge after surgery. Building the pre-admission model will proceed in stages. Three separate models will be constructed corresponding to pre-operative patient factors, procedure factors and hospital factors (see Table 5). After trimming non-significant terms, models will be compared on their residual deviance. The model with the lowest deviance will serve as the foundation, adding remaining covariates and retaining only those attaining significance at *p* < 0.05; this approach ensures retention of a reasonably small set of predictors, consistent with potential real-time incorporation into system operations when a quick result is requisite. Results will be compared to a full model containing all predictors. The readmission predictive model will be constructed by adding post-operative covariates to the pre-operative model, incorporating statistically significant post-operative variables into the final model. In addition, a full model will be estimated for comparison.

**Table 5:** Specific variables to be included in the three separate models described in aim 1

	Pre-Admission Model	Discharge Model	Enhanced Model
Variables			
Pre-Admission			
Patient Factors	X	X	X
# Pre-index admissions	X	X	X
Age	X	X	X
ASA class	X	X	X
Co-morbid conditions	X	X	X
Do Not Resuscitate status	X	X	X
Functional status	X	X	X
Gender	X	X	X
Lab values	X	X	X
Marital status	X	X	X
Pain score	X	X	X
Race	X	X	X
Smoking/Alcohol status	X	X	X
Procedure Factors	X	X	X
Fiscal Year	X	X	X
Indication for surgery	X	X	X
Operation complexity	X	X	X
Procedure type	X	X	X
Hospital Factors	X	X	X
Facility (or VISN)	X	X	X
Post-Operative/Pre-Discharge			
Surgical Complications		X	X
Hospital Acquired Infections		X	X
Emergent/Elective		X	X
Lab values		X	X
Length of Stay		X	X
Pain Score		X	X
Procedure Characteristics		X	X
Vital Signs at Discharge		X	X
Post-Discharge			
Care Coordination at Discharge			X
Caregiver Accessibility			X
Cognitive Function			X
Depression/Mood			X
Discharge Complexity			X
Discharge Destination			X
Functional Status at Discharge			X
Healthcare Utilization			X
Perceived Stress			X
Post-Discharge Complications			X
Transportation			X

#### Risk prediction tool

The starting point for constructing the risk prediction tool will be the final parsimonious pre-operative and time-of-discharge predictive models described. A second pair of pre-operative and time-of-discharge models will be developed using classification and regression trees (CART) including all available covariates. The better-performing model at each time point will be identified using split-sample validation and bootstrap replication. Each bootstrap replication will be randomly partitioned into training (80%) and testing (20%) subsets. The regression and tree models will each be fit on the training subset, and their predictions on the testing subset will be used to calculate separate c-statistics for each model. *T*-tests will assess differences in mean c-statistics between models across replications. In the event the performance of the logistic regression models is superior, the corresponding tree models will be retained for predicting readmission in cases involving missing data.

#### Development of readmission reason categories

We will use the primary diagnosis for each readmission to classify readmission reason and develop readmission categories separately for colorectal, thoracic and vascular cases. To refine categories and develop subcategories, we will calculate frequency of readmission categories for the overall cohort and by surgery. Our experience with colorectal surgery is illustrative. In analyses preparatory-to-research, 1161 patients (14%) were readmitted within 30 days following 8180 elective admissions for colorectal surgery. The top 10 primary diagnoses for readmission accounted for 58% of the overall readmissions (Table 6). After identifying the top diagnoses in our ISQ-R cohorts, we will develop broad categories, e.g., (1) surgical site infection, peritoneal abscess, wound disruption; (2) electrolyte disturbance (dehydration, acute kidney failure); (3) non-surgical site infection (urinary tract infection, *Clostridium difficile*); (4) ileus/obstruction. We will also consider secondary diagnoses as these may reveal patterns not evident from the primary diagnoses alone. The goal is to develop clinically meaningful categories that can be linked to processes of care and are potentially actionable.

**Table 6:** Top 10 principal ICD-9 readmission code following colorectal surgery

ICD-9 Category	Code	Description	*N*	(%)
Injury and poisoning	998.59	Other postoperative infection	214	(18.4)
Injury and poisoning	099.74	Digestive system complications	116	(10.0)
Endocrine, nutritional, and metabolic	276.51	Dehydration	60	(5.2)
Digestive system	560.9	Unspecified intestinal obstruction	58	(5.0)
Genitourinary system	584.9	Acute kidney failure, unspecified	53	(4.6)
Injury and poisoning	998.32	Disruption of external operation wound	43	(3.7)
Digestive system	567.22	Peritoneal abscess	41	(3.5)
Genitourinary system	599.0	Urinary tract infection	38	(3.3)
Infectious and parasitic diseases	084.5	Clostridium difficile	33	(2.8)
Injury and poisoning	998.31	Disruption of internal wound	21	(1.8)

We base this iterative process and vetting of categories on our prior work with the National Surgery Office in identifying reasons for surgical case cancellations. As part of a technical assistance project, we summarized 9528 reasons into six actionable categories that are now used in tracking and reporting surgical case cancellations [30]. Once the investigative team has developed readmission reason categories and data definitions, these will be vetted and revised with the National Surgery Office Advisory Board. The readmission reason categories will be tested in our prospective assessment of surgery patients in Aim 2.

#### Processes of care and surgical readmission for Aim 2

We developed two exploratory hypotheses to analyze our prospective cohort. Regression models will examine whether readmissions attributed to electrolyte disturbances are associated with increases in serum creatinine greater than 0.5 mg/dl from admission to discharge, and whether mean and median pain score in the 24 h prior to discharge (scale 1–10) are associated with 30-day readmission. We will assess whether additional predisposing and enabling factors are associated with: 1) 30-day readmission, 2) unplanned emergency/physician visit within 30 days, and 3) composite 30-day readmission and/or unplanned visit.

Sub-analyses will examine the associations between study outcomes and depressive symptoms (as measured by the Center for Epidemiologic Studies Depression Scale, 4-item version or CESD4) [31]. As an exploratory analysis, we will also test whether a discharge prescription for antidepressants moderates the association between CESD4 score and readmission/unplanned visit. The parsimonious model will assess odds of readmission/unplanned visit as a function of CESD4 by antidepressant (interaction term).

We will carefully examine the relationships between readmission/unplanned visit outcomes and enabling/predisposing factors in the context of the Andersen model, adding each factor to the at-discharge model from Aim 1. A final parsimonious model will result from forward-stepwise selection augmenting the at-discharge model with significant predisposing and enabling factors. The final prospective models will be developed and tested using split sample validation and bootstrapping as above, retaining the model with the best split-validation performance. Then we will determine whether the final prospective model has greater discrimination than the final retrospective at-discharge model from Aim 1.

#### Reasons for readmission – Aim 3

We will convene a Delphi panel of surgeons (general, vascular and thoracic), non-surgeon clinicians (hospitalist, general internist, emergency department physician) and quality improvement nurses to review the reasons for readmission among VASQIP-assessed colorectal, thoracic and vascular cases. The panel will rank the degree to which the reasons for readmission developed in Aims 1 and 2 are (a) potentially preventable and (b) appropriate measures of surgical quality. We will use the RAND Appropriateness Method (RAM), [32] widely used in the development of patient safety and quality improvement tools [33–35]. Our team has employed the modified RAM to identify the potential preventability of inpatient readmissions for acute myocardial infarction, heart failure and pneumonia [36, 37].

Our modified RAM consensus-building process entails these steps: 1) Define the goal of the quality measure, 2) Review the literature for similar measures, 3) Collect clinical input and data analysis to develop the measure, and 4) Consensus process - convene a panel of experts to rank each measure on a scale of appropriateness, ranging from 1 (extremely inappropriate) to 9 (extremely appropriate) [32]. We will consider each category for colorectal, thoracic and vascular cases and determine whether it incorporates preventability and poor quality of care.

We define consensus according to the RAM: all but one Delphi panelist scores within a range of 3 points around the median rank. To determine which reasons for readmission were most preventable or useful, we will use the following criteria: 1) consensus among participants, and 2) a median rating for “appropriateness as a measure of preventability for readmission” or “appropriateness as a measure of surgical quality” of 7 or higher.

At the completion of the modified Delphi process, we will have established consensus around which reasons for readmission are most likely to be potentially preventable and which reasons indicate poor surgical quality for colorectal, thoracic and vascular surgery patients.

## Discussion

The study described herein is underway. Some of the large data analyses have been completed, quantifying preoperative, intraoperative and postoperative factors as important but limited predictors of readmission (10% of variance explained) [38]. Collection of psychosocial data from the prospective cohort is underway, as is the process of understanding reasons for readmission per the panel of experts. The psychosocial predictors may prove valuable for identifying and improving management of at-risk surgical patients, and possibly discharge planning may be tailored to the patient’s medical and psychosocial status. For patients not yet readmitted prior to the follow-up outpatient appointment typically scheduled for 2–3 weeks post-discharge, failure to attend the scheduled appointment could trigger immediate outreach, as those patients could be coping with situations that could lead to long-term poor outcomes, such as inadequate transportation or other social supports impacted by their changed physical status.

## Limitations

The types of surgery studied are limited to three – colorectal, thoracic and vascular, while other types were excluded, therefore the factors identified and conclusions drawn may not be applicable to arthroscopic, orthopaedic, gynecologic, obstetric, head-and-neck, spinal, trauma-related and other types of procedures. This initial choice seemed reasonable given the higher rates of unplanned readmissions following these categories of procedures. The Delphi panel does not include non-hospital clinicians and laypeople, and therefore may fail to identify reasons that are salient to those groups or less apparent to hospital clinicians.

### Future directions

We expect that this study will lead to improved understanding of the reasons for readmission following colorectal, thoracic and vascular surgical procedures. This may allow us to develop novel quality measures based on readmissions in certain categories. Moreover, we will be able to develop and test interventions that address factors associated with preventable readmission. Future work should expand the Delphi panel to include community providers, caregivers and patients to deepen our understanding of social context, and allow clinicians to evolve a patient-centred approach to interventions for reducing readmissions. Depending on our findings, these interventions may focus on enhancing structural factors, peri-discharge practices or patient and caregiver support.

Identifying the factors that predict readmission will transition the field from the current narrow focus on operative and hospital care to an approach that pays equal attention to addressing social determinants of health and patient and caregiver knowledge, attitudes and skills. Incorporating veteran-centric care while helping our patients stay out of the hospital furthers VA’s goal to provide the highest quality care possible.

## Abbreviations

ACA: Affordable Care Act
ASA: American Society of Anesthesiologists physical status classification
BMI: Body-mass index
BUN: Blood urea nitrogen
CART: Classification and regression trees
CDW: Corporate Data Warehouse (of the VA)
CESD4: Center for Epidemiologic Studies Depression Scale, 4-item version
CMS: Centers for Medicare and Medicaid Services
CTM-15: Care Transitions Measures, with 15 items
DNR: Do not resuscitate
DVT: Deep venous thrombosis
eGFR: Estimated Glomerular Filtration Rate
ER: Emergency Room
GEE: Generalized Estimating Equations
ICD9: International Classification of Disease, 9^th^ Edition
ICU: Intensive care unit
IHI: Institute for Healthcare Improvement
INR: International Normalized Ratio
IQR: Inter-Quartile Range
ISQ-R: Improving Surgical Quality: Readmission
LOS: Length of stay
MCA: Managerial Cost Accounting (a series of VA files)
MoCA: Montreal Cognitive Assessment
PSS: Cohen’s Perceived Stress Scale
RAM: RAND Appropriateness Method
RBC: Red blood cell
RUG-ADL: Resource Utilization Group – Activities of Daily Living
RVU: Relative value units
SBT: Short blessed test
SSI: Surgical site infection
VA: Veterans Health Administration
VASQIP: Veterans Affairs Surgical Quality Improvement Program
VISN: Veterans integrated service network
VR-12: Veterans RAND 12-item health survey
WBC: White blood cell

## References

[1] Coley KC, Williams BA, DaPos SV, Chen C, Smith RB (2002). Retrospective evaluation of unanticipated admissions and readmissions after same day surgery and associated costs. J Clin Anesth.

[2] Ricciardi R, Roberts PL, Read TE, Baxter NN, Marcello PW, Schoetz DJ (2011). Mortality rate after nonelective hospital admission. Arch Surg.

[3] Patient Protection and Affordable Care Act of 2010, Pub. L. No. 111–148, 124 Stat. 119, amended by Health Care and Education Reconciliation Act of 2010, Pub. L. No. 111–152, 124 Stat. 1029 (codified as amended in scattered sections of 42 U.S.C.). 2010. https://www.gpo.gov/fdsys/pkg/PLAW-111publ152/pdf/PLAW-111publ152.pdf. Accessed 9 Mar 2017.

[4] Martin RC, Brown R, Puffer L, Block S, Callender G, Quillo A, Scoggins CR, McMasters KM (2011). Readmission rates after abdominal surgery: the role of surgeon, primary caregiver, home health, and subacute rehab. Ann Surg.

[5] Halfon P, Eggli Y, Pretre-Rohrbach I, Meylan D, Marazzi A, Burnand B (2006). Validation of the potentially avoidable hospital readmission rate as a routine indicator of the quality of hospital care. Med Care.

[6] van Walraven C, Dhalla IA, Bell C, Etchells E, Stiell IG, Zarnke K, Austin PC, Forster AJ (2011). Derivation and validation of an index to predict early death or unplanned readmission after discharge from hospital to the community. CMAJ.

[7] Bobay KL, Jerofke TA, Weiss ME, Yakusheva O (2010). Age-related differences in perception of quality of discharge teaching and readiness for hospital discharge. Geriatr Nurs.

[8] Smith DM, Giobbie-Hurder A, Weinberger M, Oddone EZ, Henderson WG, Asch DA, Ashton CM, Feussner JR, Ginier P, Huey JM (2000). Predicting non-elective hospital readmissions: a multi-site study. Department of Veterans Affairs Cooperative Study Group on Primary Care and Readmissions. J Clin Epidemiol.

[9] Garcia-Perez L, Linertova R, Lorenzo-Riera A, Vazquez-Diaz JR, Duque-Gonzalez B, Sarria-Santamera A (2011). Risk factors for hospital readmissions in elderly patients: a systematic review. QJM.

[10] Wick EC, Shore AD, Hirose K, Ibrahim AM, Gearhart SL, Efron J, Weiner JP, Makary MA (2011). Readmission rates and cost following colorectal surgery. Dis Colon Rectum.

[11] Jackson BM, Nathan DP, Doctor L, Wang GJ, Woo EY, Fairman RM (2011). Low rehospitalization rate for vascular surgery patients. J Vasc Surg.

[12] Itani KM (2009). Fifteen years of the national surgical quality improvement program in review. Am J Surg.

[13] Lucas DJ, Haider A, Haut E, Dodson R, Wolfgang CL, Ahuja N, Sweeney J, Pawlik TM (2013). Assessing readmission after general, vascular, and thoracic surgery using ACS-NSQIP. Ann Surg.

[14] Khuri SF, Daley J, Henderson W, Hur K, Demakis J, Aust JB, Chong V, Fabri PJ, Gibbs JO, Grover F (1998). The department of veterans affairs’ NSQIP: the first national, validated, outcome-based, risk-adjusted, and peer-controlled program for the measurement and enhancement of the quality of surgical care. national VA surgical quality improvement program. Ann Surg.

[15] Buck CJ (2013). 2013 ICD-9-CM for hospitals, volumes 1, 2, & 3 - professional edition.

[16] White CL, Brady TL, Saucedo LL, Motz D, Sharp J, Birnbaum LA (2015). Towards a better understanding of readmissions after stroke: partnering with stroke survivors and caregivers. J Clin Nurs.

[17] Odonkor CA, Hurst PV, Kondo N, Makary MA, Pronovost PJ (2015). Beyond the hospital gates: elucidating the interactive association of social support, depressive symptoms, and physical function with 30-day readmissions. Am J Phys Med Rehabil.

[18] Andersen RM (1995). Revisiting the behavioral model and access to medical care: does it matter?. Soc Sci Med.

[19] Vashi AA, Fox JP, Carr BG, D’Onofrio G, Pines JM, Ross JS, Gross CP (2013). Use of hospital-based acute care among patients recently discharged from the hospital. JAMA.

[20] McCusker J, Karp I, Cardin S, Durand P, Morin J (2003). Determinants of emergency department visits by older adults: a systematic review. Acad Emerg Med.

[21] Khuri SF, Daley J, Henderson W, Hur K, Gibbs JO, Barbour G, Demakis J, Irvin G, Stremple JF, Grover F (1997). Risk adjustment of the postoperative mortality rate for the comparative assessment of the quality of surgical care: results of the national veterans affairs surgical risk study. J Am Coll Surg.

[22] Noel PH, Copeland LA, Perrin RA, Lancaster AE, Pugh MJ, Wang CP, Bollinger MJ, Hazuda HP (2010). VHA corporate data warehouse height and weight data: opportunities and challenges for health services research. J Rehabil Res Dev.

[23] Price LE, Shea K, Gephart S (2015). The veterans affairs’s corporate data warehouse: uses and implications for nursing research and practice. Nurs Adm Q.

[24] Copeland LA, Zeber JE, Sako EY, Mortensen EM, Pugh MJ, Wang CP, Restrepo MI, Flynn J, MacCarthy AA, Lawrence VA (2015). Serious mental illnesses associated with receipt of surgery in retrospective analysis of patients in the Veterans Health Administration. BMC Surg.

[25] Khuri SF, Daley J, Henderson WG (2002). The comparative assessment and improvement of quality of surgical care in the Department of Veterans Affairs. Arch Surg.

[26] Szeto HC, Coleman RK, Gholami P, Hoffman BB, Goldstein MK (2002). Accuracy of computerized outpatient diagnoses in a veterans affairs general medicine clinic. Am J Manag Care.

[27] Borzecki AM, Wong AT, Hickey EC, Ash AS, Berlowitz DR (2004). Can we use automated data to assess quality of hypertension care?. Am J Manag Care.

[28] Sohn MW, Arnold N, Maynard C, Hynes DM (2006). Accuracy and completeness of mortality data in the Department of Veterans Affairs. Popul Health Metrics.

[29] Hawn MT, Graham LA, Richman JS, Itani KM, Henderson WG, Maddox TM (2013). Risk of major adverse cardiac events following noncardiac surgery in patients with coronary stents. JAMA.

[30] Argo JL, Vick CC, Graham LA, Itani KM, Bishop MJ, Hawn MT (2009). Elective surgical case cancellation in the Veterans Health Administration system: identifying areas for improvement. Am J Surg.

[31] Melchior LA, Huba GJ, Brown VB, Reback CJ (1993). A short depression index for women. Educ Psychol Meas.

[32] Fitch K, Bernstein SJ, Aguilar MS, Burnand B, LaCalle JR, Lazaro P, van het Loo M, McDonnell J, Vader JP, Kahan JP (2001). The RAND/UCLA appropriateness method user’s manual. RAND monograph report. RAND health and RAND Europe.

[33] Black N, Murphy M, Lamping D, McKee M, Sanderson C, Askham J, Marteau T (1999). Consensus development methods: a review of best practice in creating clinical guidelines. J Health Serv Res Policy.

[34] Brook RH, Chassin MR, Fink A, Solomon DH, Kosecoff J, Park RE (1986). A method for the detailed assessment of the appropriateness of medical technologies. Int J Technol Assess Health Care.

[35] Jones J, Hunter D (1995). Consensus methods for medical and health services research. BMJ.

[36] Rosen A (2011). Validating and classifying VA readmissions for quality assessment, IRR 09 369 2.

[37] Ashton CM, Kuykendall DH, Johnson ML, Wray NP, Wu L (1995). The association between the quality of inpatient care and early readmission. Ann Intern Med.

[38] Morris MS, Graham LA, Richman JS, Hollis RH, Jones CE, Wahl T, Itani KM, Mull HJ, Rosen AK, Copeland L, Burns E, Telford G, Whittle J, Wilson M, Knight SJ, Hawn MT (2016). Postoperative 30-day readmission: time to focus on what happens outside the hospital. Ann Surg.

[39] Fries BE, Schneider DP, Foley WJ, Gavazzi M, Burke R, Cornelius E (1994). Refining a case-mix measure for nursing homes: resource utilization groups (RUG-III). Med Care.

[40] McCaffery M, Beebe A (1993). Pain: clinical manual for nursing practice.

[41] Cohen S, Kamarck T, Mermelstein R (1983). A global measure of perceived stress. J Health Soc Behav.

[42] MoCATest.org. http://www.mocatest.org/. Accessed 9 Mar 2017.

[43] Vest JR, Gamm LD, Oxford BA, Gonzalez MI, Slawson KM (2010). Determinants of preventable readmissions in the United States: a systematic review. Implement Sci.

[44] Weaver C, Schiech L, Held-Warmkessel J, Kedziera P, Haney E, DiLullo G, Babb JS, Ruth K, Dell D, Barsevick A (2006). Risk for unplanned hospital readmission of patients with cancer: results of a retrospective medical record review. Oncol Nurs Forum.

[45] Coleman EA. The Care Transitions Program: Health Care Services for Improving Quality and Safety During Care Hand-Offs. University of Denver Colorado; 2012. http://caretransitions.org/. Accessed 9 Mar 2017.

[46] Rutherford P, Nielsen GA, Taylor J, Bradke P, Coleman E. How-to guide: improving transitions from the hosptial post-acute care settings to reduce avoidable rehospitalizations. Cambridge: Institute for Healthcare Improvement; 2013. Available at: http://www.ihi.org/. Accessed 9 Mar 2017.

